# Mycobacterium Fluoroquinolone Resistance Protein D (MfpD), a GTPase-Activating Protein of GTPase MfpB, Is Involved in Fluoroquinolones Potency

**DOI:** 10.1128/spectrum.02098-22

**Published:** 2022-12-01

**Authors:** Yu Huang, Shuangquan Yan, Yuzhu Li, Xuefeng Ai, Xi Yu, Yan Ge, Xi Lv, Lin Fan, Jianping Xie

**Affiliations:** a Institute of Modern Biopharmaceuticals, State Key Laboratory Breeding Base of Eco-Environment and Bio-Resource of the Three Gorges Area, Key Laboratory of Eco-Environments in Three Gorges Reservoir Region, Ministry of Education, School of Life Sciences, Southwest University, Chongqing, China; b Shanghai Clinic and Research Center of Tuberculosis, Shanghai Pulmonary Hospital, Tongji University School of Medicine, Shanghai Key Laboratory of Tuberculosis, Shanghai, China; AP-HP

**Keywords:** mycobacterial protein fragment complementation, GTPase activating protein, MfpB, MfpD, *Mycobacterium* fluoroquinolone resistance protein, small GTPase

## Abstract

Tuberculosis (TB) caused by Mycobacterium tuberculosis infection remains one of the most serious global health problems. Fluoroquinolones (FQs) are an important component of drug regimens against multidrug-resistant *tuberculosis*, but challenged by the emergence of FQ-resistant strains. Mycobacterium fluoroquinolone resistance protein A (MfpA) is a pentapeptide protein that confers resistance to FQs. MfpA is the fifth gene in the *mfp* operon among most Mycobacterium, implying other *mfp* genes might regulate the activity of MfpA. To elucidate the function of this operon, we constructed deletion mutants and rescued strains and found that MfpD is a GTPase-activating protein (GAP) involved in FQs activity. We showed that the recombinant strains overexpressing *mfpD* became more sensitive to FQs, whereas an *mfpD* deletion mutant was more resistant to FQs. By using site-directed mutagenesis and mycobacterial protein fragment complementation, we genetically demonstrated that *mfpD* participated in FQs susceptibility via directly acting on *mfpB*. We further biochemically demonstrated that MfpD was a GAP capable of stimulating the GTPase activity of MfpB. Our studies suggest that MfpD, a GAP of MfpB, is involved in MfpA-mediated FQs resistance. The function of MfpD adds new insights into the role of the *mfp* operon in Mycobacterium fluoroquinolone resistance.

**IMPORTANCE** Tuberculosis is one of the leading causes of morbidity and mortality worldwide largely due to increasingly prevalent drug-resistant strains. Fluoroquinolones are important antibiotics used for treating multidrug-resistant tuberculosis (MDR-TB). The resistance mechanism mediated by the Mycobacterium fluoroquinolone resistance protein (MfpA) is unique in Mycobacterium. However, the regulatory mechanism of MfpA remains largely unclear. In this study, we first report that MfpD acts as a GAP for MfpB and characterize a novel pathway that controls Mycobacterium small G proteins. Our findings provide new insights into the regulation of MfpA and inspiration for new candidate targets for the discovery and development of anti-TB drugs.

## INTRODUCTION

Tuberculosis (TB) caused by Mycobacterium tuberculosis (Mtb) infection claimed around 1.3 million deaths in 2020 (1). Drug-resistant TB remains a public health threat, especially multidrug-resistant (MDR)-TB, which is defined as resistant to both isoniazid and rifampicin. The treatment of MDR-TB requires multiple drugs combination, including several second-line drugs.

Fluoroquinolones (FQs) are important second-line drugs for treating MDR-TB. The well-recognized targets of FQs are type II topoisomerases, which can be divided into topoisomerases IV and DNA gyrase (2). DNA gyrase is the only type II topoisomerase present in M. tuberculosis (3). The DNA gyrase is an essential enzyme involved in key cellular processes, including DNA replication, transcription, and stress responses (4). FQs inhibit gyrase by forming a DNA-DNA gyrase-fluoroquinolone ternary complex. The formation of ternary complex arrests the DNA replication machinery at blocked replication forks, resulting in DNA synthesis (at lower FQs concentrations), inhibition, and cell death (at lethal concentrations) (5, 6).

The most common mechanism of M. tuberculosis high-level quinolone resistance is mutations within the quinolone resistance determining regions (QRDRs) of its DNA gyrase encoding genes (7). Mutations in the QRDRs of gyrase alter its affinity for FQs. The amino acid substitutions conferring resistance in the QRDR-A (codons 74 to 113) occur most commonly in codons 88, 90, 91, and 94 (8). Mutations in QRDR-B (codons 500 to 538) have also been associated with FQs resistance and most often occur in codons 500 and 538 (9). To interact with DNA gyrase, FQs must penetrate the cell envelope, and mutations that result in the reduction of intracellular concentration, either by increasing efflux or decreasing uptake, also confer resistance (10, 11). Resistance mediated by MfpA, which belongs to pentapeptide repeat proteins (PRPs) (12), is a rather new finding.

PRPs, consisting of tandemly repeated amino acid sequences with a consensus sequence of [S,T,A,V][D,N][L,F][S,T,R][G] (13), can confer resistance to FQs (14). A Mycobacterium PRP homolog, namely, MfpA, was found by screening a genomic library for genes conferring resistance to FQs. The MICs of M. smegmatis recombinants with MfpA expressed on plasmids to all FQs increased 2- to 8-fold (12). Structure showed that the binding of MfpA with DNA gyrase prevented the formation of DNA-DNA gyrase-FQs ternary complex (15). Recent results showed that MfpA bound to the GyrB subunit of gyrase and stimulated its ATPase activity to relieve gyrase from FQs inhibition (16). However, the regulation of *mfpA* is unknown. In most sequenced Mycobacterium species, *mfpA* is located in an operon, preceded by four conserved genes (17). In M. tuberculosis, four conserved proteins are named MfpB to MfpE, followed by MfpA. The fourth gene, *Rv3362c*, encodes MfpB, a small GTPase that was reported to be involved in MfpA’s protection of DNA gyrase (18). The third gene, *Rv3363c*, encodes MfpC, a protein of 122 amino acids belonging to the DUF742 family whose function is unclear. The second gene, *Rv3364c*, encodes MfpD, a 130-amino-acids peptide that belongs to the Roadblock/LC7 family. MglB, a Roadblock/LC7 protein in Myxococcus xanthus, was a GTPase-activating protein (GAP) of an adjacent GTPase, MglA (19, 20). The first gene, *Rv3365c*, encodes MfpE, which is similar to histidine kinases and may serve as a sensor. For the function of the *mfp* operon, MfpE may sense some unknown signal then is transmitted to the effector MfpA through the GAP MfpD that stimulates the GTPase activity of MfpB (17). However, to our knowledge, no direct experimental evidence supports this hypothesis except MfpB.

To explore the role of MfpD in the regulation of MfpA and its relationship with FQs susceptibility, we analyzed the effects of MfpD *in vivo* using M. smegmatis as a model. We found that MfpD was involved in FQs susceptibility. Gene deletion experiments and M-PFC analyses (21) indicated that MfpD directly interacted with GTPase MfpB. Further experiments with purified MfpD and MfpB proteins showed that MfpD was a GAP and it could increase the GTP hydrolysis activity of MfpB. Collectively, our results revealed that MfpD was a GAP and its interaction with MfpB could alter MfpA-mediated FQs resistance.

## RESULTS

### MfpD increases susceptibility to fluoroquinolone *in vivo*.

**FIG 1:**
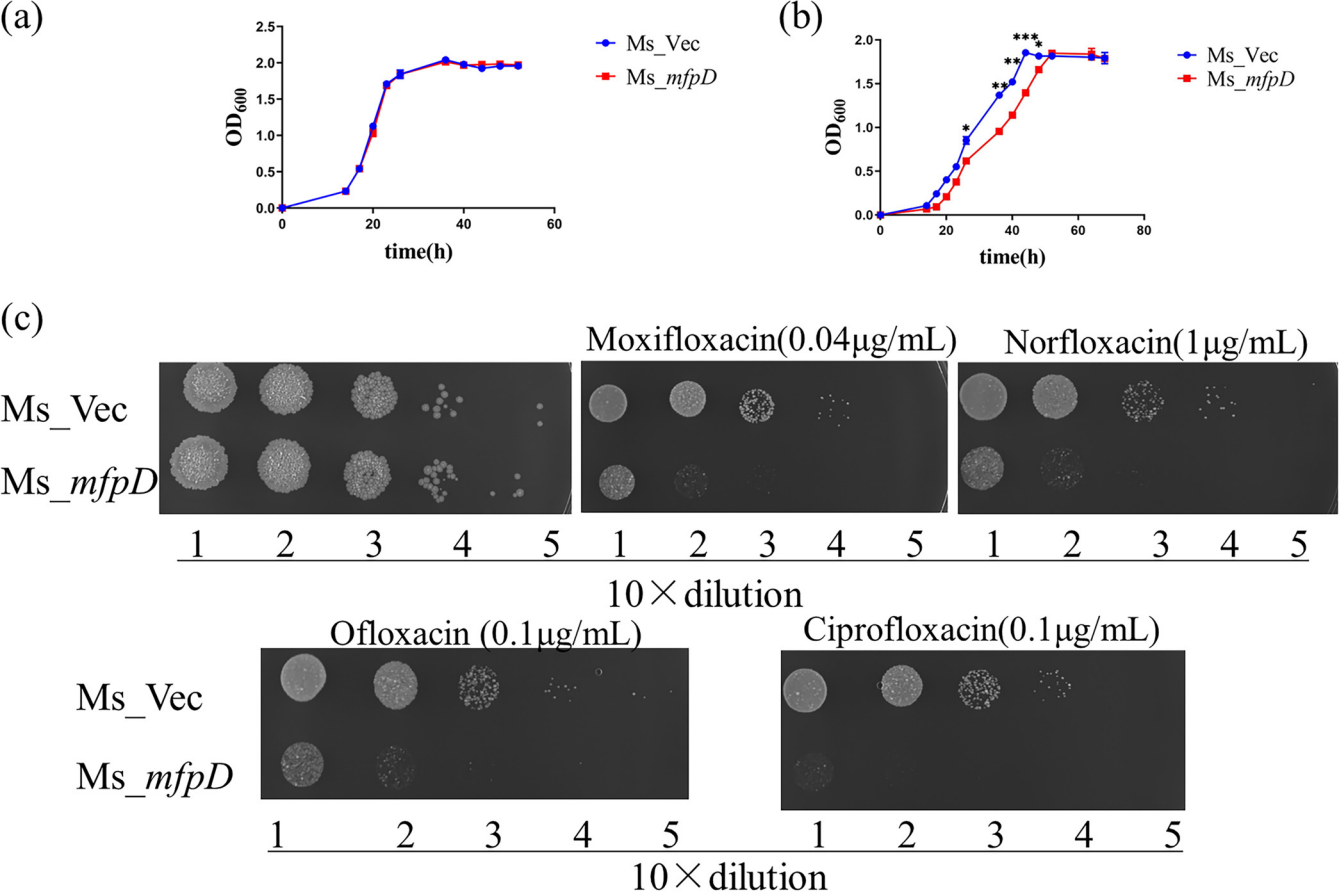
The overexpression of *mfpD* enhances susceptibility to FQs. (a and b) Growth of Ms_Vec and Ms_*mfpD*. Ms_Vec and Ms_*mfpD* were grown in 7H9 medium (a) supplemented with 0.03 μg/mL moxifloxacin (b). (c) Growth of Ms_Vec and Ms_*mfpD* under fluoroquinolones exposure. Tenfold serial dilutions of Ms_Vec and Ms_*mfpD* were spotted on 7H10 supplemented with moxifloxacin, norfloxacin, ciprofloxacin, and ofloxacin. The result was recorded after 3 days incubation.

To test whether MfpD was involved in the M. smegmatis susceptibility to FQs, we constructed M. smegmatis recombinant strains Ms_Vec and Ms_*mfpD* (overexpression of *mfpD*). The growth between Ms_Vec and Ms_*mfpD* was almost identical, suggesting that *mfpD* had no or negligible effect on the growth of M. smegmatis (Fig. 1a). Next, we examined the growth of the recombinant strains in the presence of 0.04 μg/mL moxifloxacin. At this concentration, the growth of Ms*_mfpD* was lagged, whereas the Ms_Vec grew normally (Fig. 1b). Moreover, the growth of recombinant strains was examined in different FQs by spotting cells on the 7H10 medium. The Ms*_mfpD* strain became more susceptible in the presence of different FQs (Fig. 1c). Our data indicated that the overexpression of *mfpD* could increase susceptibility to FQs *in vivo*.

**FIG 2:**
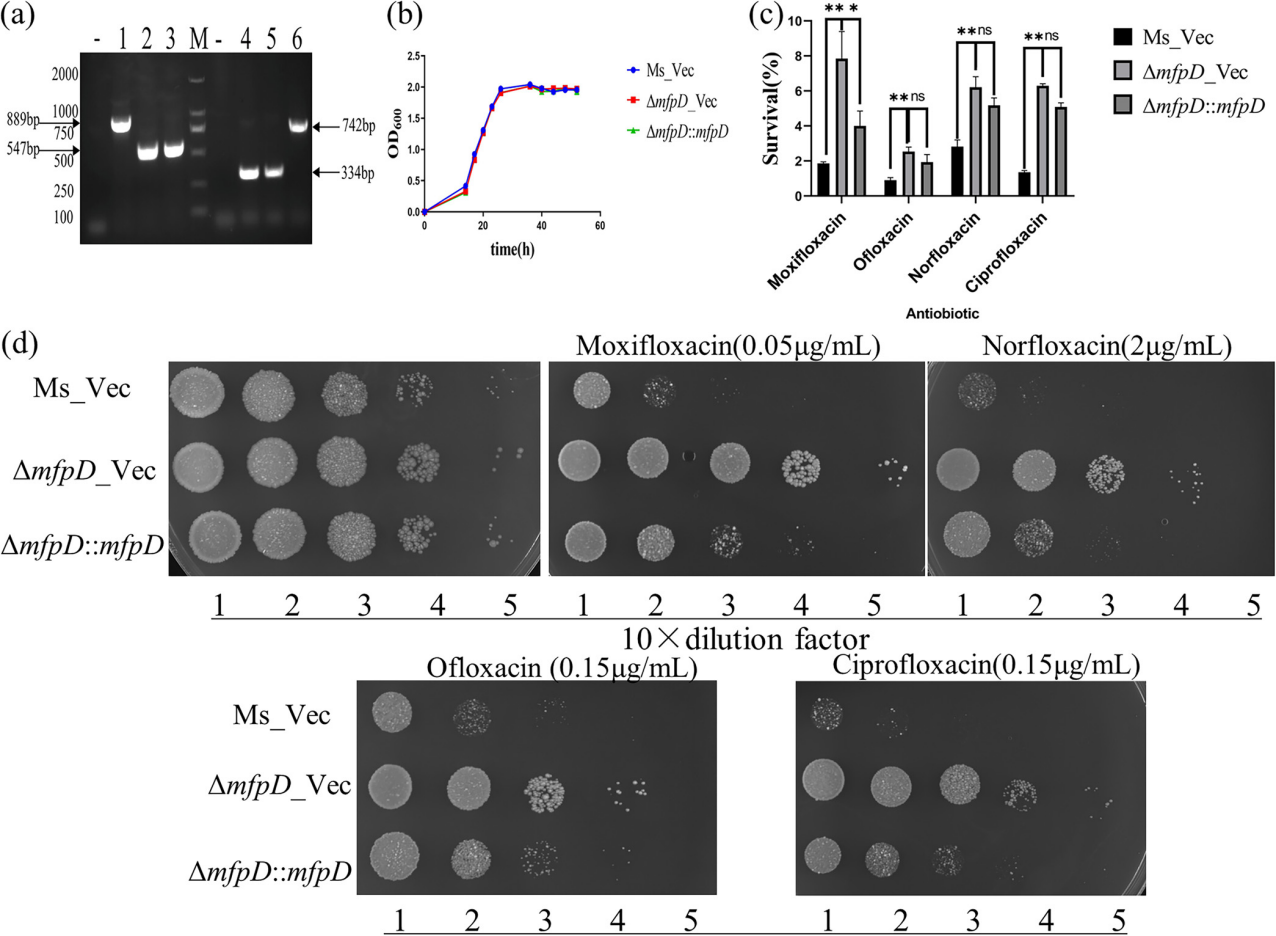
The deficiency of *mfpD* decreases susceptibility to FQs. (a) PCR amplification of *mfpD* in Ms_Vec (lane 1), Δ*mfpD_*Vec (lane 2), and Δ*mfpD*::*mfpD* (lane 3) by using 37–38 (up) and 38–39 (down) primers; PCR amplification of pMV-261 in Ms_Vec (lane 4), Δ*mfpD_*Vec (lane 5), and Δ*mfpD*::*mfpD* (lane 6) by using pMV-F and pMV-R primers. M, DL2000; -, negative control. (b) Growth of Ms_Vec, Δ*mfpD*_Vec, and Δ*mfpD*::*mfpD*. Strains were grown in 7H9 medium supplemented. (c) Cells were treated with different FQs. After 3 h treatment, the CFU was recorded, and the survival calculated (%). (d) Growth of Ms_Vec, Δ*mfpD*_Vec, and Δ*mfpD*::*mfpD* under FQs exposure. Tenfold serial dilutions of strains were spotted on the 7H10 plate supplemented with moxifloxacin, norfloxacin, ofloxacin, and ciprofloxacin. The result was recorded after 3 days incubation at 37°C.

To test whether *mfpD* can alter the growth of the strain, an *mfpD*-deletion mutant (Δ*mfpD*) and its complemented strain (Δ*mfpD*::*mfpD*) were also constructed (Fig. 2a). The growth result showed that the deletion of *mfpD* did not affect the growth of M. smegmatis (Fig. 2b). Then, we examined the effect of *mfpD* deficiency on the lethality of FQs (Fig. 2c). The results showed that the survival of Δ*mfpD* was increased, and the survival was decreased when the wild-type *mfpD* copy was introduced to Δ*mfpD*. Also, cells were spotted on 7H10 plates supplemented with different FQs (Fig. 2d). The Δ*mfpD* is less susceptible to FQs than Ms_Vec, and the phenotype could be partially reversed by complementation. Next, constructed strains were treated with novobiocin, which targets GyrB, another subunit of gyrase. We did not observe any growth difference among Ms_Vec, Δ*mfpD*_Vec, and Δ*mfpD*::*mfpD* (Fig. S1 in the supplemental material). Taken together, these results showed that MfpD was involved in Mycobacterium fluroquinolone susceptibility.

### Mycobacterium *mfp* operon genes are cotranscribed.

**FIG 3:**
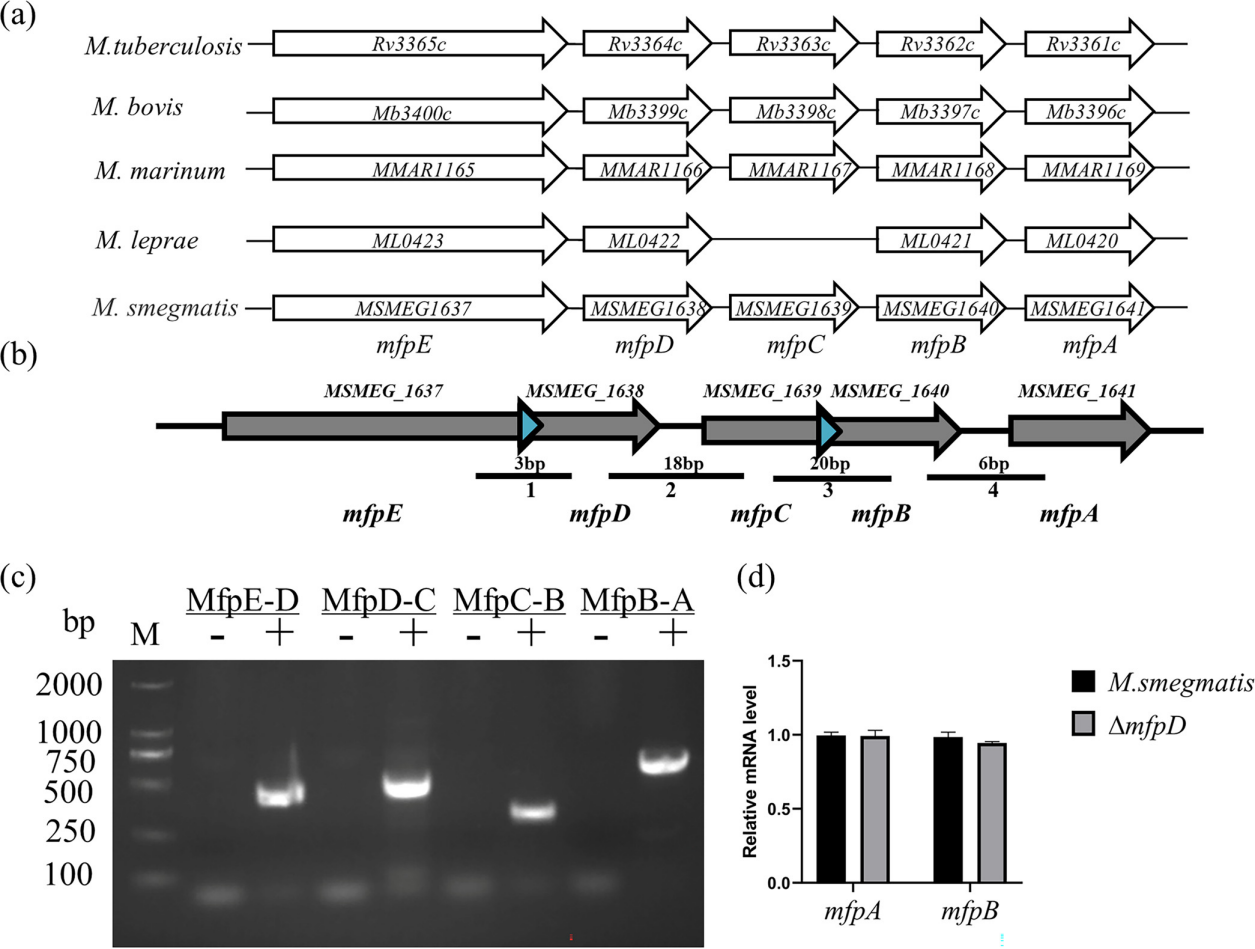
The *mfp* operon is cotranscribed. (a) Sequence and structural characteristics of the Mycobacterium *mfp* operon. (b) The schematic diagram of primers in RT-PCR analysis. Black arrows represent the genes of the *mfp* operon, and blue arrows represent the overlapping area of gene pairs. Black bars represent the PCR-amplified intergenic regions of the *mfp* operon. (c) RT-PCR analysis of the *mfp* operon. M, DL2000; -, negative controls; +, cDNA as the template. (d) The mRNA level of *mfpB* and *mfpA* in Δ*mfpD* and M. smegmatis.

To further determine the role of *mfpD* in the *mfp* operon, the conservation of the *mfp* operon was analyzed (Fig. 3a). The result indicated that the *mfp* operon was highly conserved in Mycobacterium. The *mfp* operon was cotranscribed in Mycobacterium (22); we repeated the cotranscription experiment to prove that the susceptibility caused by *mfpD* was related to *mfpA*. The reverse transcription (RT)-PCR was used to amplify the intergenic regions of the operon (Fig. 3b). cDNAs were synthesized from M. smegmatis RNAs using a reverse transcriptase kit. Negative control was conducted by precluding the reverse transcriptase enzyme during RT reactions (Fig. 3c). We observed that PCR fragments could be amplified by each primer pair. The results indicated that the *Mfp* operon was cotranscribed in Mycobacterium. Because *mfpD* and *mfpA* were cotranscribed, the phenotype of *mfpD* might be caused by the polar effect. To explore this possibility, the mRNA levels of *mfpA* and *mfpB* were tested in M. smegmatis and Δ*mfpD* (Fig. 3d). The results indicated that the deficiency of *mfpD* did not significantly change *mfpA* and *mfpB* transcription. In summary, the phenotype of *mfpD* is not caused by the polar effect.

### MfpD acts in the same pathways as MfpB.

**FIG 4:**
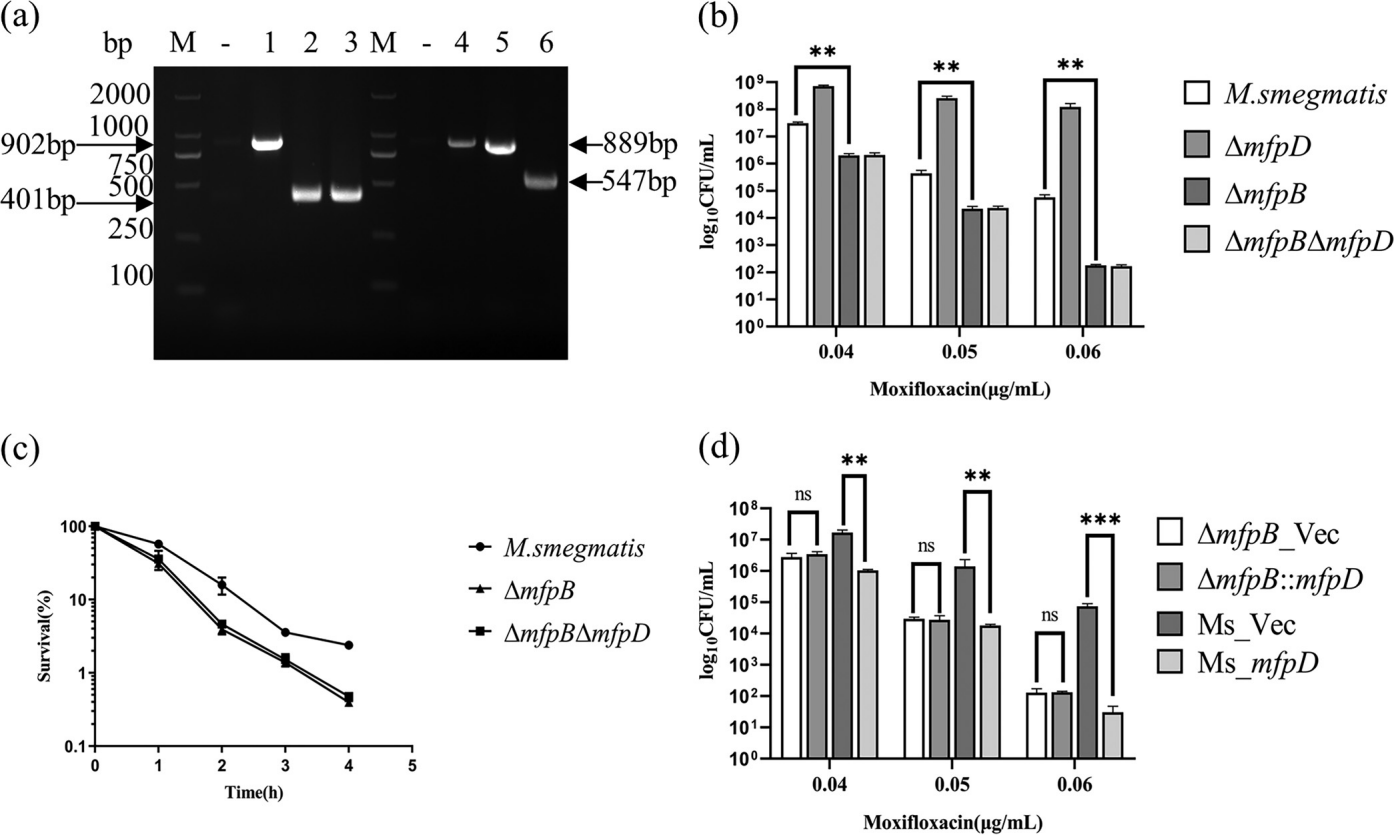
MfpD acts in the same pathways as MfpB. (a) PCR amplification of *mfpB* in M. smegmatis (lane 1), Δ*mfpB* (lane 2), and Δ*mfpB*Δ*mfpD* (lane 3) by using 39–40 (up) and 40–41 (down) primers; PCR amplification of *mfpD* in M. smegmatis (lane 4), Δ*mfpB* (lane 5), and Δ*mfpB*Δ*mfpD* (lane 6) by using 37–38 (up) and 38–39 (down) primers. M, DL2000; -, negative controls. (b) Growth of strains upon exposure to moxifloxacin. Tenfold serial dilutions of strains were spotted on 7H10 containing moxifloxacin. The result was recorded after 3 days incubation at 37°C. (c) Cells were treated with moxifloxacin. After indicated times, the CFU was recorded and the survival calculated (%). (d) Growth of Δ*mfpB*_Vec, Δ*mfpB*::*mfpD*, Ms_Vec, and Ms_*mfpD* under moxifloxacin pressure.

Since genes in the *mfp* operon were cotranscribed, we speculated that MfpD might alter the Mycobacterium fluoroquinolone susceptibility by regulating its downstream gene(s). Previous studies reported that MfpB could help MfpA to protect gyrase from FQs (18). We hypothesized that MfpD might act in the same pathways as MfpB. Epistasis tests were performed to test this hypothesis. Firstly, Δ*mfpB* and Δ*mfpB*Δ*mfpD* mutants were constructed (Fig. 4a) and cultured in 7H9 or 7H10 medium supplemented with moxifloxacin. Δ*mfpB* was more sensitive to moxifloxacin, consistent with previous reports (18). Compared with Δ*mfpD*, Δ*mfpB*Δ*mfpD* was more sensitive and had the same phenotype as Δ*mfpB* (Fig. 4b and c). The results indicated that the phenotype effect of *mfpD* was only evident in the presence of *mfpB*. To further corroborate this conclusion, pMV-261-*mfpD* was transformed into Δ*mfpB*, then cells were cultured on 7H10 plates containing moxifloxacin (Fig. 4d). Consistent with our prediction, MfpD failed to alter moxifloxacin sensitivity in Δ*mfpB*, while MfpD could increase the M. smegmatis sensitivity to FQs. Thus, the results showed that MfpD acts in the same pathways as MfpB, and MfpD is epistatic on MfpB.

### MfpD interacts directly with MfpB.

**FIG 5:**
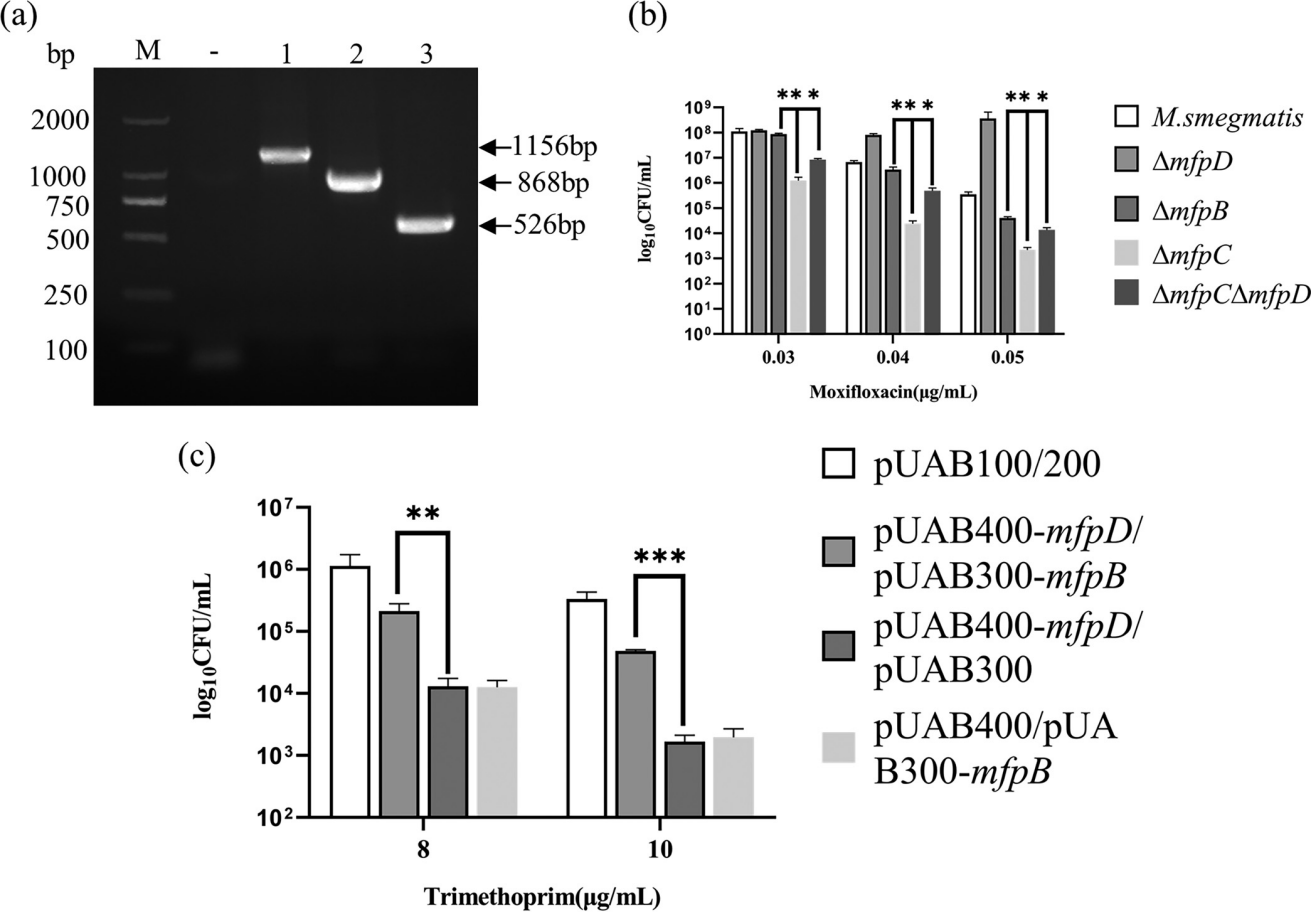
MfpD interacts with MfpB directly. (a) PCR amplification in M. smegmatis (lane 1), Δ*mfpC* (lane 2), and Δ*mfpC*Δ*mfpD* (lane 3) by using 38–39 (up) and 39–40 (down) primers. M, DL2000; -, negative controls. (b) Growth of cells on 7H10 supplemented with moxifloxacin. (c) M-PFC analysis demonstrated that MfpD interacted with MfpB directly. pUAB400-*mfpD*/pUAB300-*mfpB*, positive control (cells contained pUAB100 and pUAB200), and negative controls (cells contained pUAB400-*mfpD* and pUAB300; and pUAB400 and pUAB300-*mfpB*) were cultured on 7H10 plates supplemented with TRIM. The result was recorded after 3 days incubation at 37°C.

Next, we hypothesized that MfpD interacted with MfpB directly. To test this educated guess, we first ruled out the possibility that MfpD affected MfpB by acting on MfpC. Also, Δ*mfpC* and Δ*mfpC*Δ*mfpD* mutants were constructed (Fig. 5a) and cultured in 7H10 medium supplemented with moxifloxacin (Fig. 5b). Compared with M. smegmatis, Δ*mfpC* became hypersensitive (more sensitive than Δ*mfpB*) to moxifloxacin, and the susceptibility level of Δ*mfpC*Δ*mfpD* was between Δ*mfpD* and Δ*mfpC*. This phenomenon precluded a regulatory relationship between MfpD and MfpC, and supported that MfpD acted in pathways different from MfpC.

To further determine that MfpD interacted directly with MfpB *in vivo*, M-PFC (mycobacterial protein fragment complementation) was used to study protein–protein interaction by estimating trimethoprim (TRIM) susceptibility. Briefly, the coding regions of *mfpD* and *mfpB* were cloned into pUAB400 and pUAB300, respectively, to construct pUAB400-*mfpD* and pUAB300-*mfpB*. Plasmid pairs were cotransformed into M. smegmatis. pUAB400-*mfpD/*pUAB300-*mfpB*, positive control (cells contained plasmid pairs pUAB100 and pUAB200), and negative control (cells contained plasmid pairs pUAB400-*mfpD* and pUAB300; and pUAB400 and pUAB300-*mfpB*) were cultured on 7H10 medium supplemented with TRIM (Fig. 5c). The significant difference was observed in CFU between pUAB400-*mfpD*/pUAB300-*mfpB* and negative controls, indicating that MfpD interacted with MfpB directly. Furthermore, these observations support that MfpD is involved in MfpA-mediated fluoroquinolone resistance by affecting MfpB directly.

### MfpD is a GAP for MfpB.

**FIG 6:**
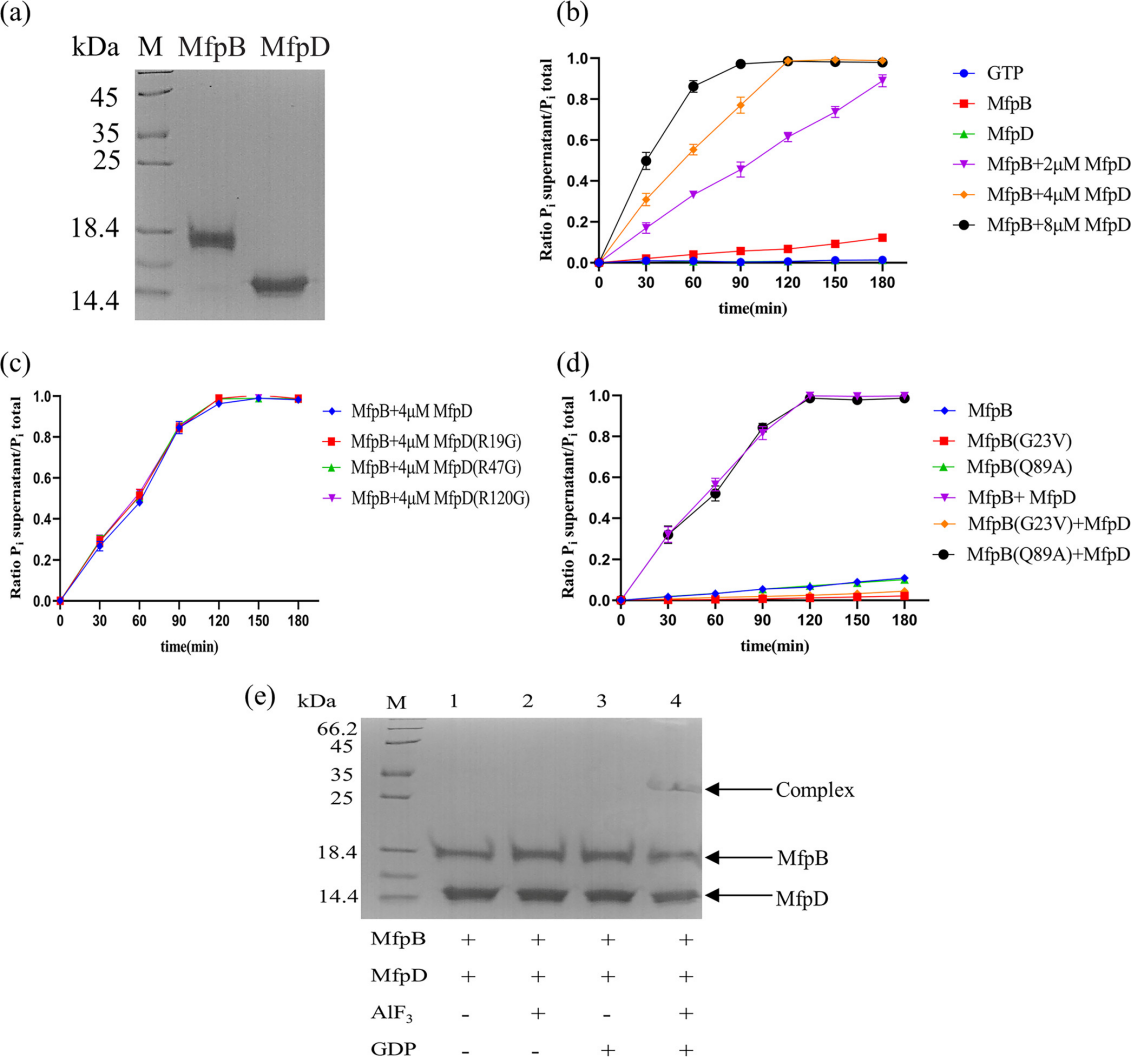
MfpD is a GAP for MfpB. (a) Purification of MfpB and MfpD. (b) MfpD stimulates the intrinsic GTPase activity of MfpB. (c) The MfpD arginine mutations cannot change the acceleration effect. (d) The MfpB G23V mutation reduces GTP hydrolysis. Graph depicts the release of Pi using a malachite green assay in the presence of 40 μM GTP and 4 μM MfpB or mutation proteins with and without the addition of MfpD as indicated. (e) MfpD binds to GDP-MfpB in the presence of AlF_3_.

In eukaryotes, the GDP-GTP cycle of small G-proteins is regulated by guanine nucleotide exchange factors (GEFs) that induce the release of the GDP to be replaced by GTP and by GAPs that stimulate the low intrinsic GTPase activity (23). In Mycobacterium, MfpB is a small GTPase and MfpD has been predicted to be a member of Roadblock/LC7 family protein (17, 18). MglB, a Roadblock/LC7 protein in Myxococcus xanthus, is a GAP of its adjacent GTPase MglA (19, 20, 24). Based on the homology between MfpD and MglB, MfpD might act as a GAP of MfpB. To test this hypothesis, MfpD and MfpB proteins were purified from recombinant E. coli (Fig. 6a). To test whether MfpD could accelerate the GTPase reaction of MfpB, the GTP hydrolysis of MfpB was analyzed in the presence or absence of MfpD. *In vitro*, the GTP hydrolysis rate of MfpB was very slow and MfpD failed to hydrolyze GTP (Fig. 6b). Moreover, the slow intrinsic GTP-hydrolysis rate of MfpB was accelerated by MfpD, and the acceleration effect was dose dependent (Fig. 6b). GAPs accelerate GTP hydrolysis by correctly positioning catalytic residues to the GTPase, providing catalytic residues directly into the active site, or a combination of both. In general, most GAPs stabilize the position of catalytic residues of small GTPase by the arginine finger (23). To test the role of the arginine finger, we mutated potentially arginine residues of MfpD, including R19G, R47G, and R120G (Fig. S2). The result showed that the acceleration effect was almost identical between arginine mutations and MfpD (Fig. 6c).

The effect of mutations in eukaryotic small GTPases such as Ras has been well characterized (25). Therefore, two MfpB mutations were generated based on the alignments with other Ras GTPases (Fig. S3). G23V mutation (equivalent to G12V in Ras) and Q89A (equivalent to Q61A in Ras) are predicted to be permanently in the GTP-bound active state because both the intrinsic and the GAP-stimulated GTPase are dramatically reduced. The GTP-hydrolysis rate of MfpB (G23V) was reduced about 8-fold, and the addition of MfpD failed to stimulate hydrolysis (Fig. 6d). Q89A mutation affected neither GTP hydrolysis nor stimulation of GTP hydrolysis by MfpD (Fig. 6d), which was different from eukaryotic Ras and Myxococcus xanthus MglA but similar to eukaryotic Rab (26).

GDP-bound G proteins require their GAP to bind to AlF_3_, and vice versa; GAPs bind to GDP-bound G proteins with high affinity only in the presence of AlF_3_ (27). As we have shown above, MfpD can accelerate the GTPase activity of MfpB, consistent with the hypothesis that MfpD is a GAP for MfpB. To further support the hypothesis, we tested whether MfpD formed a complex with GDP-bound MfpB in the presence of AlF_3_ to confirm MfpD was the GAP for MfpB (Fig. 6e). No complex formation between MfpD and MfpB was observed (lane 1). The addition of AlF_3_ or GDP alone to the MfpD and MfpB mixture didn’t result in complex formation (lanes 2 and 3). However, the addition of AlF_3_ and GDP to the mixture resulted in complex formation (lane 4). These results strongly supported that MfpD is MfpB’s GAP.

### MfpA is the effector of MfpB.

**FIG 7:**
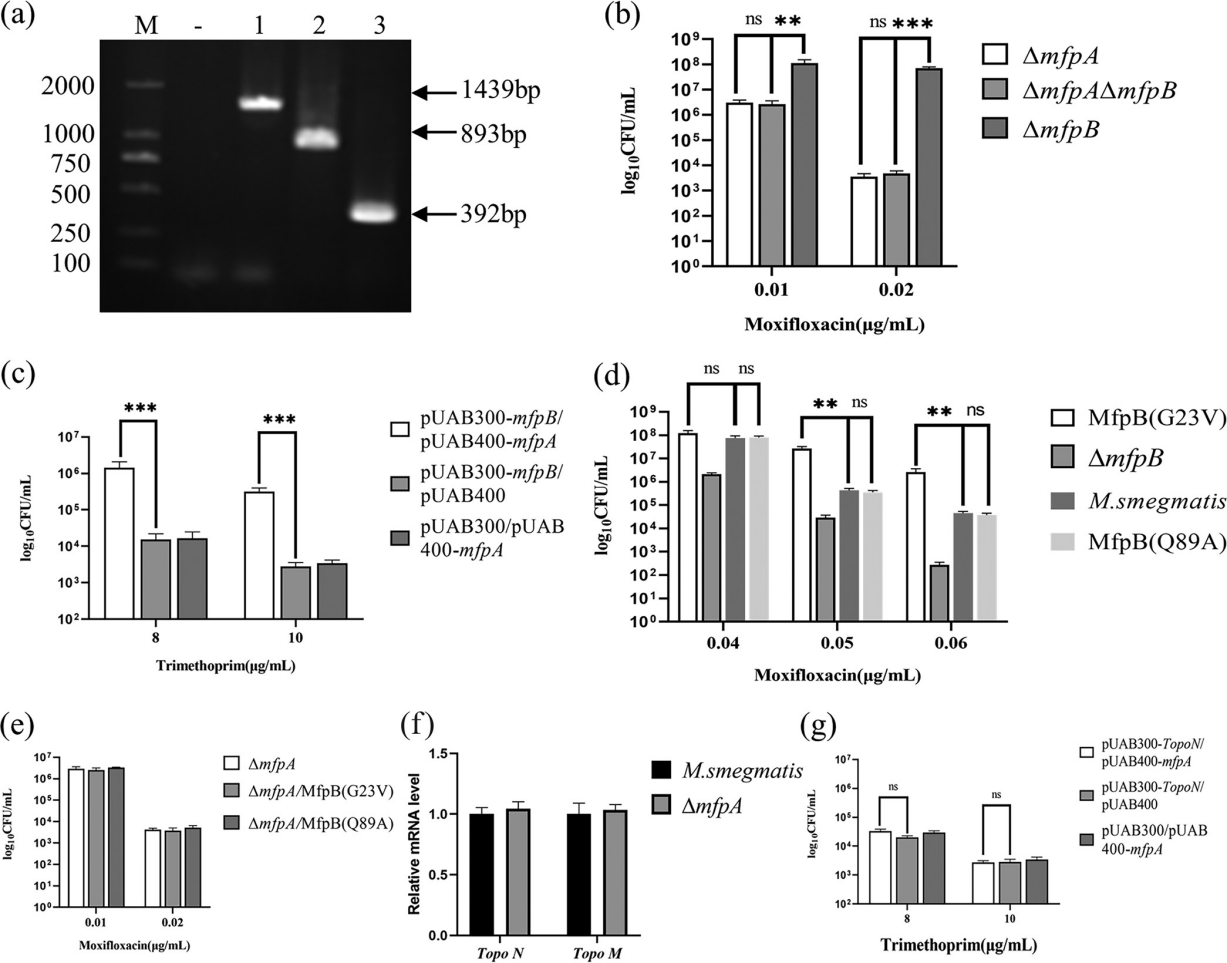
MfpA serves as an effector of MfpB. (a) PCR amplification in M. smegmatis (lane 1), Δ*mfpA* (lane 2), and Δ*mfpA*Δ*mfpB* (lane 3) by using 39–40 (up) and 1,641 (+132) primers. M, DL2000; -, negative controls. (b, d, and e) Growth of cells on 7H10 supplemented with moxifloxacin. The result was recorded after 3 days incubation at 37°C. (c and g) M-PFC analysis demonstrated that MfpB interacted with MfpA directly, and MfpA did not interact with Topo N. (f) The mRNA level of Topo N and Topo M in Δ*mfpA* and *M. smegmatis*.

MfpB was reported to facilitate MfpA to protect gyrase against FQs, but with negligible effect *in vitro* (16). To reconcile this paradox, Δ*mfpA* and Δ*mfpA*Δ*mfpB* were constructed (Fig. 7a) and cultured in 7H10 medium supplemented with moxifloxacin (Fig. 7b). Compared with Δ*mfpB*, Δ*mfpA*Δ*mfpB* was more sensitive to moxifloxacin, with susceptibility level identical to Δ*mfpA*. Besides, the M-PFC assay demonstrated that MfpA interacted directly with MfpB *in vivo* (Fig. 7c). These results indicated that the FQs phenotype of MfpB was caused by affecting MfpA *in vivo*.

Also, MfpB (G23V) and MfpB (Q89A) mutants in the native chromosomal site were constructed and tested for their effects. MfpB (G23V) was more resistant to FQs, while MfpB (Q89A) showed similar susceptibility level with M. smegmatis (Fig. 7d). The mutant results, which corresponded to the GTPase mutation assay, indicated that MfpB acted as a small G-protein switch to participate in MfpA-mediated FQs resistance. Furthermore, to test for effects between MfpA and MfpB, MfpB point mutants were constructed in Δ*mfpA*. The phenotype effect of the MfpB (G23V) mutant was only significant in the presence of *mfpA*, indicating that MfpB is epistatic on MfpA (Fig. 7e). Taken together, these results show that MfpA serves as an effector of MfpB *in vivo*.

Since M. smegmatis had an atypical type II topoisomerase Topo NM (28), the potential implication of Topo NM should be considered in the study. The potential implications of Topo NM were divided into two points: one point was the susceptibility to FQs, and another point was the interaction with MfpA. Due to Topo NM being less inhibited by FQs, which were 40-fold less than gyrase (28), the first point was not our consideration. To test for a possible relationship between MfpA and Top NM, levels of Top NM mRNA were examined in M. smegmatis and Δ*mfpA* (Fig. 7f). Levels of Topo NM mRNA were similar in M. smegmatis and Δ*mfpA*, indicating that *mfpA* did not affect Topo NM expression. In addition, previous results reported that MfpA interacted with MsgyrB47 (codons 1 to 427), the ATPase domain of GyrB (16). To test whether MfpA interacted with Topo NM *in vivo*, the ATPase domain of Topo N was cloned into pUAB300 and cotransformed with pUAB400-*mfpA* (Fig. 7g). No statistically significant difference in CFU numbers between pUAB300-*Topo N*/pUAB400-*mfpA* and the negative control indicated that *mfpA* did not interact with Topo NM. In summary, the data rule out the potential implications of Topo NM in the *mfpA* regulation scheme.

## DISCUSSION

In this study, we elucidated the molecular details of MfpD in the regulation of FQs susceptibility using M. smegmatis proteins. When *mfpD* was overexpressed, the strain became more susceptible to FQs (Fig. 1), while Δ*mfpD* was more resistant to FQs (Fig. 2). The *mfp* operon was cotranscribed (Fig. 3), suggesting that MfpD was involved in MfpA protection DNA gyrase against FQs. Genetic analyses and M-PFC showed that MfpD interacted with MfpB directly and affected MfpA-mediated FQs resistance *in vivo* (Fig. 4 and 5). Biochemical results showed the Roadblock/LC7 family protein MfpD was a GAP for MfpB (Fig. 6).

**FIG 8:**
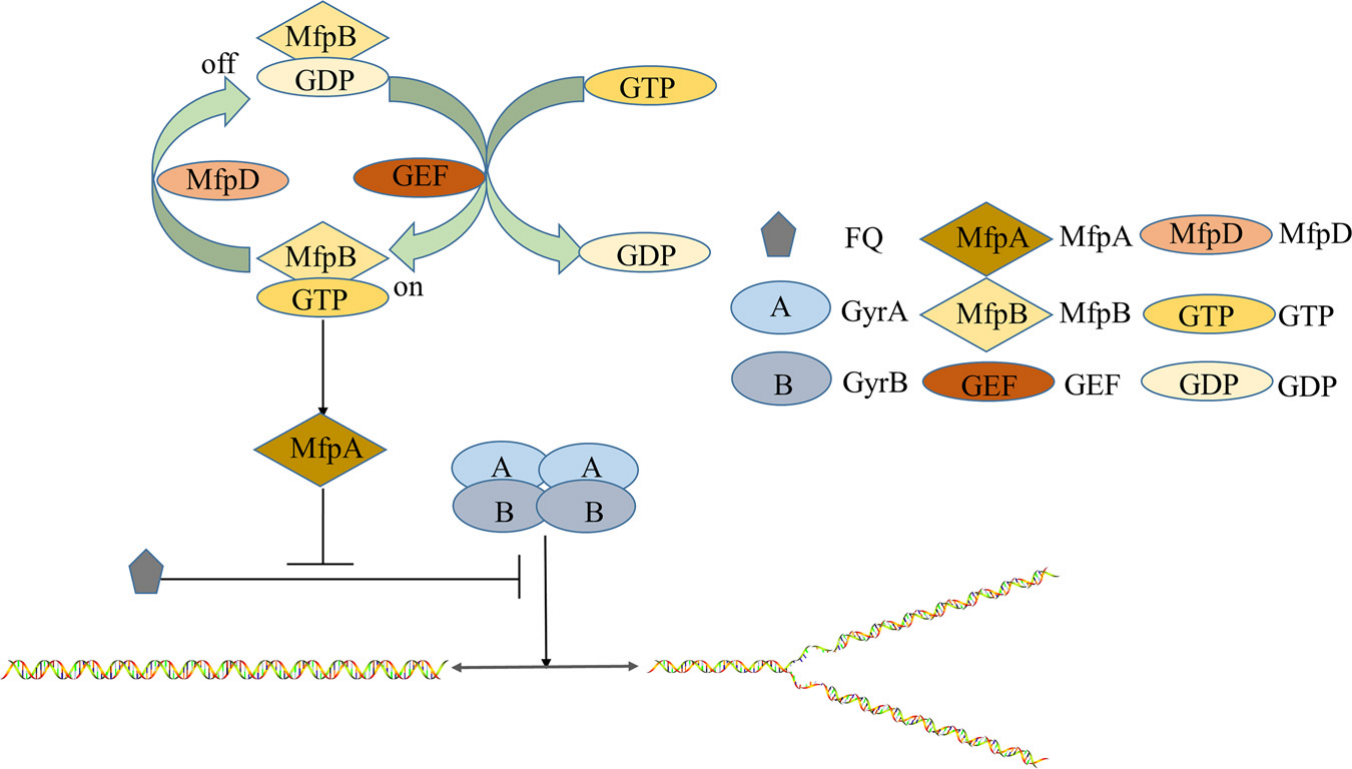
Role of MfpD in MfpA-mediated FQs resistance in Mycobacterium. The deficiency of MfpD leads to the accumulation of GTP-bound MfpB, which will make more MfpA in active form. The more active form of MfpA can inhibit the formation of a DNA-DNA gyrase-fluoroquinolone ternary complex, and protect DNA gyrase against fluoroquinolone.

Based on the above observations, we propose a model to interpret how MfpD functions in MfpA-mediated FQs resistance. First, a GAP can enhance the GTPase activity of its G protein, and converts the G protein from the GTP bound to the GDP bound (23). We have proven that MfpD was a GAP for MfpB, and MfpA was an effector of MfpB (Fig. 7). Δ*mfpD* abolishes GTPase stimulation activity and causes the GTP-bound MfpB to accumulate *in vivo*. MfpB (G23V), with GTPase activity reduced, is locked in GTP-bound form. The increase of GTP-bound MfpB makes more MfpA in the active form. The active form of MfpA will inhibit the formation of the DNA-DNA gyrase-fluoroquinolones ternary complex and protect DNA gyrase against FQs (Fig. 8).

In prokaryotes, most GTPases are involved in translation, signal transduction, cell motility, and assembly of the maturation of ribosomal subunits (29). For example, FtsZ participates in the regulation of bacterial division (30), Obg attributes to DNA replication and ribosome maturation (31), and HflX controls hypoxia-induced replication arrest in slow-growing Mycobacterium (32). During the GTPase cycle, small G proteins are tightly regulated by GAPs, GEFs, and guanine nucleotide dissociation inhibitors (GDIs) (33). Although regulation of small G proteins is ubiquitous in eukaryotes, it is rare in prokaryotes. YihI was identified as the first prokaryotic GAP, stimulating the GTPase activity of Der (34). In M. xanthus, MglB was a GAP of MglA, and RomR-RomX acted as a GEF of MglA to regulate its activity (35). To the best of our knowledge, this is the first report about a function of a GAP protein MfpD that regulates its cognate G protein and FQs susceptibility in Mycobacterium.

We established that MfpD is the GAP of MfpB. However, many questions remain. For instance, conflicting evidence about whether MfpB is necessary for MfpA protection *in vivo* and *in vitro* suggests that other factors may be involved in MfpA-mediated FQs resistance. Although we have found that MfpA was the effector of MfpB by genetic assays, more biochemical evidence is needed.

The next question is how MfpD performs its GAP function. Most GAPs regulate cognate G protein by providing Arg finger. However, mutations in conserved Arg of MfpD do not change its acceleration activity. Previous studies mentioned that Arg53 in MglA, a homolog of MfpB, was the potential substitute of the Arg-finger GAPs. It is interesting to explore whether Arg62 of MfpB (Fig. S4) plays a role in GAP-stimulated GTP hydrolysis (24). Also, according to the crystal structure results of the MglA/MglB complex, Ala62 and Ala66 in MfpD (Fig. S5) are potential residues that affect complex formation. Moreover, the crystal structures of the MfpD/MfpB complex would help to provide a greater understanding of its mechanism.

Finally, though the *mfp* operon is cotranscribed, the upregulation of *mfpD* and the upregulation of *mfpA* or *mfpB* have opposite effects on resistance to FQs. Other latent regulation elements may be present in this operon. In addition, the affinity of MfpB for GTP/GDP is in the micromole range (K_d_ ≈ 0.01 μM [18]). The direct consequence of this high affinity is a slow dissociation of nucleotides, and activation of G proteins in biological processes happens within minutes or even less, which requires the activity of GEFs (23). Moreover, GAPs usually coexist with GEFs, and whether MfpC is a GEF of MfpB remains to be explored.

In summary, we have shown that MfpD, a GAP of MfpB, cooperates with MfpA to protect DNA gyrase from FQs damage. The MfpD–MfpB interaction can be targeted for new chemical entities against *Mycobacteria*.

## MATERIALS AND METHODS

### Bacterial strains, plasmid, primer, and growth conditions.

M. smegmatis and other constructed strains were grown in Middlebrook 7H9 medium supplemented with 0.2% glycerol and 0.05% Tween 80 or Middlebrook 7H10 medium supplemented with 0.5% glycerol. Luria-Bertani medium was used to culture E. coli strains. Kanamycin (25 μg/mL for M. smegmatis, 50 μg/mL for E. coli) and hygromycin (50 μg/mL for M. smegmatis, 150 μg/mL for E. coli) were added to the medium. All plasmids, primers, and strains used in this study are listed in Table S1.

### Construction of knockout mutant strains, complementation, and overexpression strains.

All mutants were constructed by CRISPR-Cas12a (Cpf1)-assisted recombination (36). Here, we took Δ*mfpD* as an example and briefly described its construction process. The upstream region of *mfpD* was amplified with the mfpDP1/P2 primer pair, and the downstream region of *mfpD* was amplified with the mfpDP3/P4 primer pair. Then their PCR products were recovered and overlapped to amplify homologous fragments by using the mfpDP1/P4 primer pair. The crmfpDup/dw primer pair was annealed and then cloned into pCR-Hyg plasmid to construct crRNA expression plasmids. Homologous fragments (500 ng to 700ng) and crRNA plasmid (300 ng to 500ng) were mixed and then electroporated into M. smegmatis that harbored pJV53-Cpf1 plasmid. The electroporated cells were plated and grown on 7H10 medium supplemented with kanamycin, hygromycin, and 50 ng/mL anhydrotetracycline (the inducer of Cpf1). After growth for 4 to 5 days at 30°C, transformants were cultured and confirmed by PCR and sequencing analysis. The coding region of *mfpD* was amplified from M. smegmatis genomic DNA and cloned into the vector pMV-261 to generate pMV-261-*mfpD*. Then, pMV-261-*mfpD* was electroporated into mutant or M. smegmatis, generating complementation or overexpression strains.

### Antibiotic susceptibility assays.

Three methods were used for measuring the antibiotics effect.

**Spot tests.** Strains were grown to OD_600_ = 0.8 to 1.0 and tested for their susceptibility to antibiotics by spotting a 10-fold serial dilution on 7H10 medium supplemented with different antibiotics: moxifloxacin, ofloxacin, norfloxacin, and ciprofloxacin.

**Growth curve.** Strains in the log phase were diluted 10-fold and cultured in 7H9 medium supplemented with antibiotics. The OD_600_ was determined at an interval of 4 h. After culturing for about 3 days, GraphPad Prism software was used to draw curves. Three parallels were required for each strain.

**Survival curve.** Mid-exponential-phase cells were diluted and cultured in 7H9 medium supplemented with the indicated antibiotics. Surviving cells were determined by CFU on the 7H10 medium. The percentage surviving was determined by calculating CFU recovered relative to an untreated sample at the time points when antibiotics were added. Three replicates were performed for each strain.

### Reverse transcription (RT)-PCR.

RT-PCR was used to test whether the *mfp* operon was cotranscribed. Total RNA was extracted from M. smegmatis according to the manufacturer’s instructions (Tiangen, China). A reverse transcription kit (Takara) was used to synthesize the cDNA. Negative control was prepared to exclude DNA genomic pollution. The only difference in negative control was the absence of reverse transcriptase when using a reverse transcription kit. Then, cDNA and negative control were used to amplify the target band to verify gene cotranscription.

### Mycobacterial protein fragment complementation (M-PFC).

The association between proteins *in vivo* was assessed using M-PFC (21). Here, the interaction between MfpD and MfpB was taken as an example. Briefly, the coding regions of *mfpD* and *mfpB* were cloned into pUAB400 and pUAB300 to generate pUAB400-*mfpD* and pUAB300-*mfpB*, respectively. pUAB400-*mfpD*/pUAB300 and pUAB400/pUAB300-*mfpB* plasmid pairs were used as negative control; the pUAB100/pUAB200 plasmid pair was used as a positive control. The above plasmid pairs were respectively cotransformed into M. smegmatis by electroporation. All transformants were cultured on 7H10 supplemented with trimethoprim (TRIM) to confirm the interaction of MfpD and MfpB. Also, M-PFC was used to confirm the interaction between MfpB and MfpA, MfpA, and Topo N.

### The purification of MfpD and MfpB.

MfpB and MfpD were expressed separately from modified pET-28a vectors containing an N-terminal hexahistidine SUMO tag, using BL21 *E.coli* cells. Substitutions of amino acids in MfpB were introduced into the *mfpB* coding region using overlapping PCR. Recombinant strains were induced by 1 mM IPTG, followed by incubation at 16°C overnight. Cells were harvested by centrifugation at 10,000 rpm for 20 min and resuspended in different buffers according to subsequent experiments (HEPES buffer was used to determine GTPase activity, and Tris-HCl buffer was used in aluminum fluoride experiments). Then, cells were lysed by sonication for 40 min (sonication cycle was 3 s on and 2 s off). Lysates were centrifuged at 10,000 rpm, 4°C to remove debris before purification. Then, supernatants were incubated with Ni-NTA agarose for 2 h at 4°C. The beads were washed with a washing buffer in which the concentration of imidazole was 20 mM for 5 column volumes. Proteins were eluted with elution buffer in which the concentration of imidazole was 250 mM. Purified proteins were detected by SDS-PAGE. SUMO-tagged protein was then cleaved by SUMO protease and dialyzed overnight to remove imidazole. The cleaved protein was passed over the Ni-NTA column, and the flowthrough was then collected and concentrated. HEPES buffer: 25 mM HEPES (pH 7.5), 150 mM NaCl. Tris-HCl buffer: 20 mM Tris-HCl (pH 7.5), 150 mM NaCl.

### Measurement of the GTPase activity.

The GTPase activity of the protein was determined using the ammonium molybdate assay (37). Briefly, MfpB (4 μM) or point mutation was incubated in 25 mM HEPES buffer (pH 6.8), which contained 50 mM NaCl and 5 mM MgCl_2_ without and with different concentrations of MfpD for 10 min. Then, 40 μM GTP was added to the mixtures and the mixtures were incubated at 37°C. Every 30 min, solution (160 μL) was taken from the mixtures. Forty microliters of color reagent (2.5 mL of 7.5% ammonium molybdate was added to 10 mL of the dye solution followed by 0.2 mL of 11% Tween 20) was mixed with the solution and analyzed by measuring its absorbance at 650 nm. The dye solution was prepared by adding 0.44g malachite green to 360 mL sulfuric acid (3.1 M). The standard curve used to consult the conversion of Pi content and absorbance value was determined by measuring KH_2_PO_4_ as the substrate.

### Aluminum fluoride experiment.

A total of 1 mg MfpB and 1.5 mg MfpD were mixed in Buffer M (50 mM Tris-HCl, pH = 8.0, 10% glycerol, 50 mM NaCl, 25 mM MgCl_2_, 1 mM DTT). Twenty millimolars of GDP and AlF_3_ were added to mixtures according to the experimental group. Mixtures (200 μL) were incubated on ice overnight. SDS-PAGE was used to confirm the formation of the complex.

### Statistical analysis.

The data were analyzed using Student's *t* test. Significance was defined as *P* values (***, *P < *0.001; **, *P < *0.01; *, *P < *0.05).

## References

[1] WHO. 2021. Global tuberculosis report 2020. World Health Organization, Geneva, Switzerland.

[2] Schoeffler AJ, Berger JM. 2008. DNA topoisomerases: harnessing and constraining energy to govern chromosome topology. Q Rev Biophys 41:41–101. doi:10.1017/S003358350800468X. 18755053

[3] Cole ST, Brosch R, Parkhill J, Garnier T, Churcher C, Harris D, Gordon SV, Eiglmeier K, Gas S, Barry CE, III, Tekaia F, Badcock K, Basham D, Brown D, Chillingworth T, Connor R, Davies R, Devlin K, Feltwell T, Gentles S, Hamlin N, Holroyd S, Hornsby T, Jagels K, Krogh A, McLean J, Moule S, Murphy L, Oliver K, Osborne J, Quail MA, Rajandream MA, Rogers J, Rutter S, Seeger K, Skelton J, Squares R, Squares S, Sulston JE, Taylor K, Whitehead S, Barrell BG. 1998. Deciphering the biology of *Mycobacterium tuberculosis* from the complete genome sequence. Nature 393:537–544. doi:10.1038/31159. 9634230

[4] Gellert M, Mizuuchi K, O'Dea MH, Nash HA. 1976. DNA gyrase: an enzyme that introduces superhelical turns into DNA. Proc Natl Acad Sci USA 73:3872–3876. doi:10.1073/pnas.73.11.3872. 186775 PMC431247

[5] Drlica K. 1999. Mechanism of fluoroquinolone action. Curr Opin Microbiol 2:504–508. doi:10.1016/s1369-5274(99)00008-9. 10508721

[6] Kohanski MA, Dwyer DJ, Collins JJ. 2010. How antibiotics kill bacteria: from targets to networks. Nat Rev Microbiol 8:423–435. doi:10.1038/nrmicro2333. 20440275 PMC2896384

[7] Takiff HE, Salazar L, Guerrero C, Philipp W, Huang WM, Kreiswirth B, Cole ST, Jacobs WR, Jr, Telenti A. 1994. Cloning and nucleotide sequence of Mycobacterium tuberculosis gyrA and gyrB genes and detection of quinolone resistance mutations. Antimicrob Agents Chemother 38:773–780. doi:10.1128/AAC.38.4.773. 8031045 PMC284541

[8] Yin X, Yu Z. 2010. Mutation characterization of *gyrA* and *gyrB* genes in levofloxacin-resistant *Mycobacterium tuberculosis* clinical isolates from Guangdong Province in China. J Infect 61:150–154. doi:10.1016/j.jinf.2010.05.001. 20452372

[9] Maruri F, Sterling TR, Kaiga AW, Blackman A, van der Heijden YF, Mayer C, Cambau E, Aubry A. 2012. A systematic review of gyrase mutations associated with fluoroquinolone-resistant *Mycobacterium tuberculosis* and a proposed gyrase numbering system. J Antimicrob Chemother 67:819–831. doi:10.1093/jac/dkr566. 22279180 PMC3299416

[10] Danilchanka O, Pavlenok M, Niederweis M. 2008. Role of porins for uptake of antibiotics by *Mycobacterium smegmatis*. Antimicrob Agents Chemother 52:3127–3134. doi:10.1128/AAC.00239-08. 18559650 PMC2533485

[11] Pasca MR, Guglierame P, Arcesi F, Bellinzoni M, De Rossi E, Riccardi G. 2004. Rv2686c-Rv2687c-Rv2688c, an ABC fluoroquinolone efflux pump in *Mycobacterium tuberculosis*. Antimicrob Agents Chemother 48:3175–3178. doi:10.1128/AAC.48.8.3175-3178.2004. 15273144 PMC478549

[12] Montero C, Mateu G, Rodriguez R, Takiff H. 2001. Intrinsic resistance of *Mycobacterium smegmatis* to fluoroquinolones may be influenced by new pentapeptide protein MfpA. Antimicrob Agents Chemother 45:3387–3392. doi:10.1128/AAC.45.12.3387-3392.2001. 11709313 PMC90842

[13] Vetting MW, Hegde SS, Fajardo JE, Fiser A, Roderick SL, Takiff HE, Blanchard JS. 2006. Pentapeptide repeat proteins. Biochemistry 45:1–10. doi:10.1021/bi052130w. 16388575 PMC2566302

[14] Tran JH, Jacoby GA. 2002. Mechanism of plasmid-mediated quinolone resistance. Proc Natl Acad Sci USA 99:5638–5642. doi:10.1073/pnas.082092899. 11943863 PMC122823

[15] Hegde SS, Vetting MW, Roderick SL, Mitchenall LA, Maxwell A, Takiff HE, Blanchard JS. 2005. A fluoroquinolone resistance protein from *Mycobacterium tuberculosis* that mimics DNA. Science 308:1480–1483. doi:10.1126/science.1110699. 15933203

[16] Feng L, Mundy JEA, Stevenson CEM, Mitchenall LA, Lawson DM, Mi K, Maxwell A. 2021. The pentapeptide-repeat protein, MfpA, interacts with mycobacterial DNA gyrase as a DNA T-segment mimic. Proc Natl Acad Sci USA 118:e2016705118. doi:10.1073/pnas.2016705118. 33836580 PMC7980463

[17] Mayer C, Takiff H. 2014. The molecular genetics of fluoroquinolone resistance in *Mycobacterium tuberculosis*. Microbiol Spectr 2:MGM2-0009–2013. 10.1128/microbiolspec.MGM2-0009-2013 26104201

[18] Tao J, Han J, Wu H, Hu X, Deng J, Fleming J, Maxwell A, Bi L, Mi K. 2013. Mycobacterium fluoroquinolone resistance protein B, a novel small GTPase, is involved in the regulation of DNA gyrase and drug resistance. Nucleic Acids Res 41:2370–2381. doi:10.1093/nar/gks1351. 23275532 PMC3575795

[19] Leonardy S, Miertzschke M, Bulyha I, Sperling E, Wittinghofer A, Sogaard-Andersen L. 2010. Regulation of dynamic polarity switching in bacteria by a Ras-like G-protein and its cognate GAP. EMBO J 29:2276–2289. doi:10.1038/emboj.2010.114. 20543819 PMC2910265

[20] Zhang Y, Franco M, Ducret A, Mignot T. 2010. A bacterial Ras-like small GTP-binding protein and its cognate GAP establish a dynamic spatial polarity axis to control directed motility. PLoS Biol 8:e1000430. doi:10.1371/journal.pbio.1000430. 20652021 PMC2907295

[21] Singh A, Mai D, Kumar A, Steyn AJ. 2006. Dissecting virulence pathways of *Mycobacterium tuberculosis* through protein–protein association. Proc Natl Acad Sci USA 103:11346–11351. doi:10.1073/pnas.0602817103. 16844784 PMC1544089

[22] Falco A, Aranaga C, Ocampo I, Takiff H. 2021. Overexpression of *mfp*A gene increases ciprofloxacin resistance in *Mycobacterium smegmatis*. Int J Microbiol 2021:6689186. doi:10.1155/2021/6689186. 33824663 PMC8007378

[23] Bos JL, Rehmann H, Wittinghofer A. 2007. GEFs and GAPs: critical elements in the control of small G proteins. Cell 129:865–877. doi:10.1016/j.cell.2007.05.018. 17540168

[24] Miertzschke M, Koerner C, Vetter IR, Keilberg D, Hot E, Leonardy S, Sogaard-Andersen L, Wittinghofer A. 2011. Structural analysis of the Ras-like G protein MglA and its cognate GAP MglB and implications for bacterial polarity. EMBO J 30:4185–4197. doi:10.1038/emboj.2011.291. 21847100 PMC3199381

[25] Gremer L, Gilsbach B, Ahmadian MR, Wittinghofer A. 2008. Fluoride complexes of oncogenic Ras mutants to study the Ras-RasGap interaction. Biol Chem 389:1163–1171. doi:10.1515/BC.2008.132. 18713003

[26] Pan X, Eathiraj S, Munson M, Lambright DG. 2006. TBC-domain GAPs for Rab GTPases accelerate GTP hydrolysis by a dual-finger mechanism. Nature 442:303–306. doi:10.1038/nature04847. 16855591

[27] Wittinghofer A. 1997. Signaling mechanistics: aluminum fluoride for molecule of the year. Curr Biol 7:R682–R685. doi:10.1016/s0960-9822(06)00355-1. 9382787

[28] Jain P, Nagaraja V. 2005. An atypical type II topoisomerase from *Mycobacterium smegmatis* with positive supercoiling activity. Mol Microbiol 58:1392–1405. doi:10.1111/j.1365-2958.2005.04908.x. 16313624

[29] Verstraeten N, Fauvart M, Versees W, Michiels J. 2011. The universally conserved prokaryotic GTPases. Microbiol Mol Biol Rev 75:507–542. doi:10.1128/MMBR.00009-11. 21885683 PMC3165542

[30] Bhattacharya D, Sinha K, Panda D. 2018. Mutation of G51 in SepF impairs FtsZ assembly promoting ability of SepF and retards the division of *Mycobacterium smegmatis* cells. Biochem J 475:2473–2489. doi:10.1042/BCJ20180281. 30006469

[31] Kint C, Verstraeten N, Hofkens J, Fauvart M, Michiels J. 2014. Bacterial Obg proteins: GTPases at the nexus of protein and DNA synthesis. Crit Rev Microbiol 40:207–224. doi:10.3109/1040841X.2013.776510. 23537324

[32] Ngan JYG, Pasunooti S, Tse W, Meng W, Ngan SFC, Jia H, Lin JQ, Ng SW, Jaafa MT, Cho SLS, Lim J, Koh HQV, Abdul GN, Pethe K, Sze SK, Lescar J, Alonso S. 2021. HflX is a GTPase that controls hypoxia-induced replication arrest in slow-growing mycobacteria. Proc Natl Acad Sci USA 118:e2006717118. doi:10.1073/pnas.2006717118. 33723035 PMC8000101

[33] Ghosh P, Rangamani P, Kufareva I. 2017. The GAPs, GEFs, GDIs and…now, GEMs: new kids on the heterotrimeric G protein signaling block. Cell Cycle 16:607–612. doi:10.1080/15384101.2017.1282584. 28287365 PMC5397260

[34] Hwang J, Inouye M. 2010. A bacterial GAP-like protein, YihI, regulating the GTPase of Der, an essential GTP-binding protein in *Escherichia coli*. J Mol Biol 399:759–772. doi:10.1016/j.jmb.2010.04.040. 20434458

[35] Szadkowski D, Harms A, Carreira LAM, Wigbers M, Potapova A, Wuichet K, Keilberg D, Gerland U, Sogaard-Andersen L. 2019. Spatial control of the GTPase MglA by localized RomR–RomX GEF and MglB GAP activities enables *Myxococcus xanthus* motility. Nat Microbiol 4:1344–1355. doi:10.1038/s41564-019-0451-4. 31110363

[36] Yan MY, Yan HQ, Ren GX, Zhao JP, Guo XP, Sun YC. 2017. CRISPR-Cas12a-assisted recombineering in bacteria. Appl Environ Microbiol 83:e00947-17. doi:10.1128/AEM.00947-17. 28646112 PMC5561284

[37] Baykov AA, Evtushenko OA, Avaeva SM. 1988. A malachite green procedure for orthophosphate determination and its use in alkaline phosphatase-based enzyme immunoassay. Anal Biochem 171:266–270. doi:10.1016/0003-2697(88)90484-8. 3044186

