# Supplementary material for: Mycobacterium Fluoroquinolone Resistance Protein D (MfpD), a GTPase-Activating Protein of GTPase MfpB, Is Involved in Fluoroquinolones Potency
Source: Microbiol Spectr. 2022 Dec 1;10(6):e02098-22. doi: 10.1128/spectrum.02098-22 (PMC9769811; doi:10.1128/spectrum.02098-22)
Supplement: Supplemental file 1 — Supplemental material. Download spectrum.02098-22-s0001.pdf, PDF file, 1.3 MB [file spectrum.02098-22-s0001.pdf]

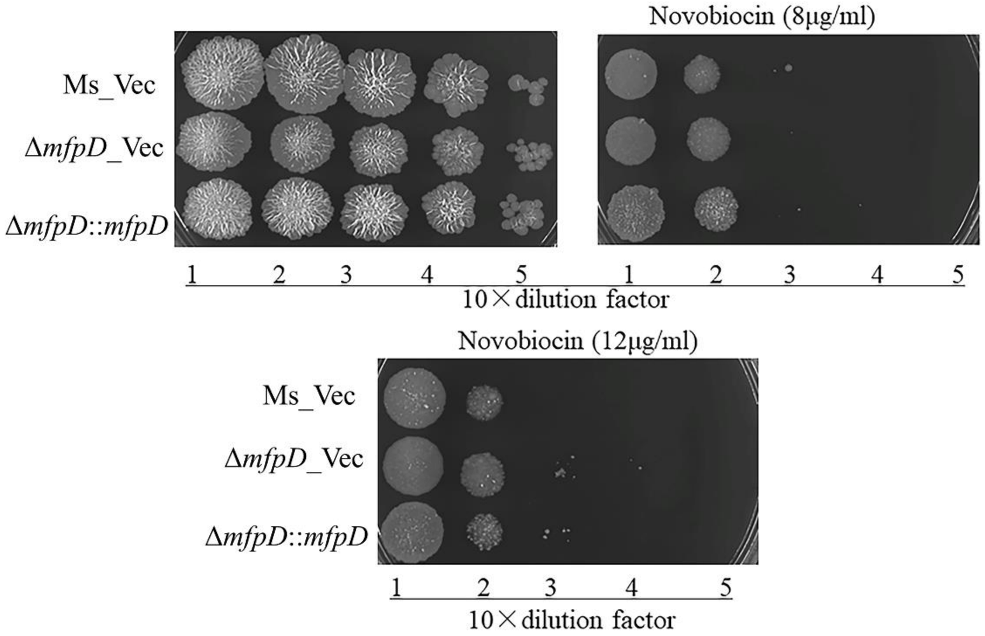

Figure S1. The deficiency of *mfpD* can't affect *M.smegmatis* resistance to Novobiocin. Growth of Ms_Vec, Δ*mfpD*_Vec and Δ*mfpD*::*mfpD* under Nocobiocin exposure. The result was recorded after 3 days incubation at 37℃.

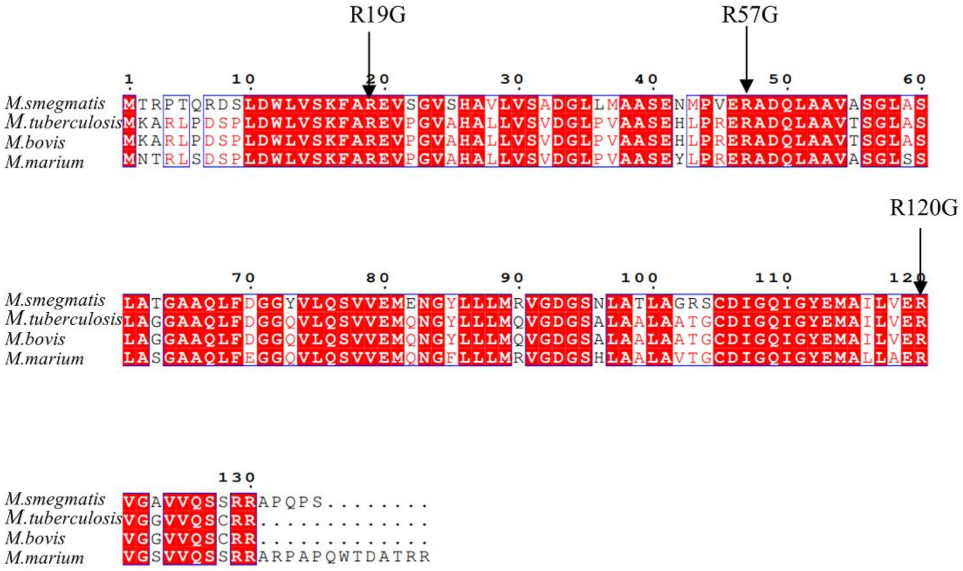

Figure S2.Sequence alignment of MfpD in *Mycobacterium*.

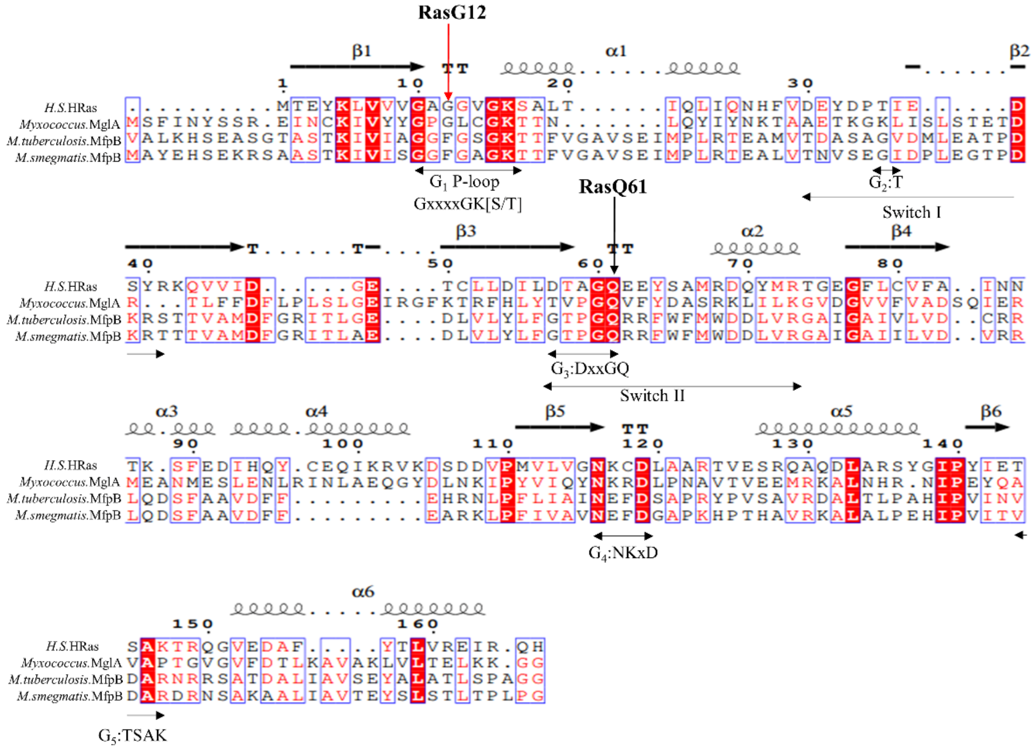

Figure S3. Multiple sequence alignment of MfpB from *Mycobacterium* and its homologs in H*omo sapiens* and *Myxococcus xanthus*.

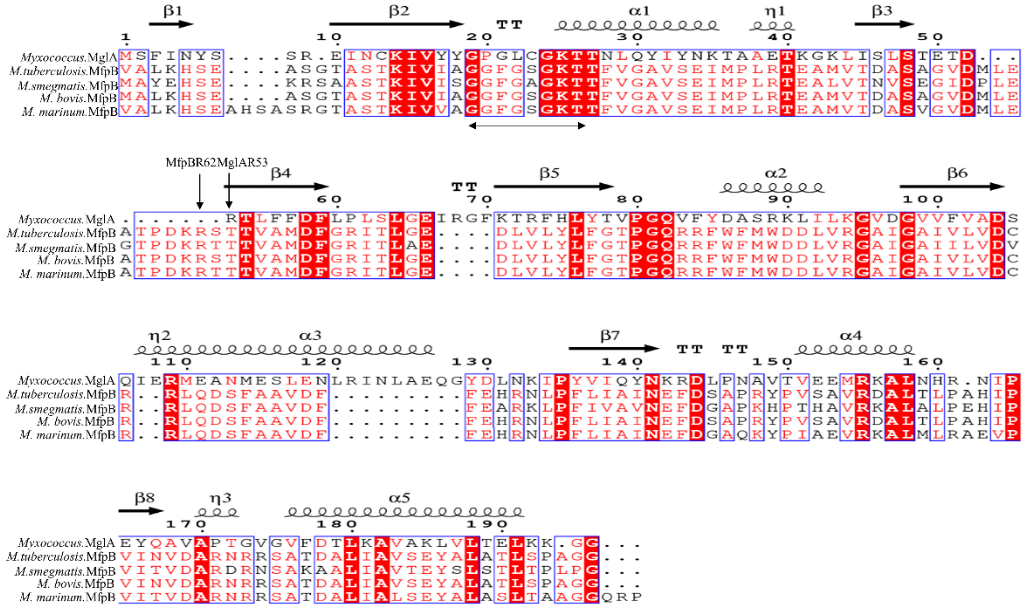

Figure S4. Multiple sequence alignment of MfpB from *Mycobacterium* and its homologs in *Myxococcus xanthus*.

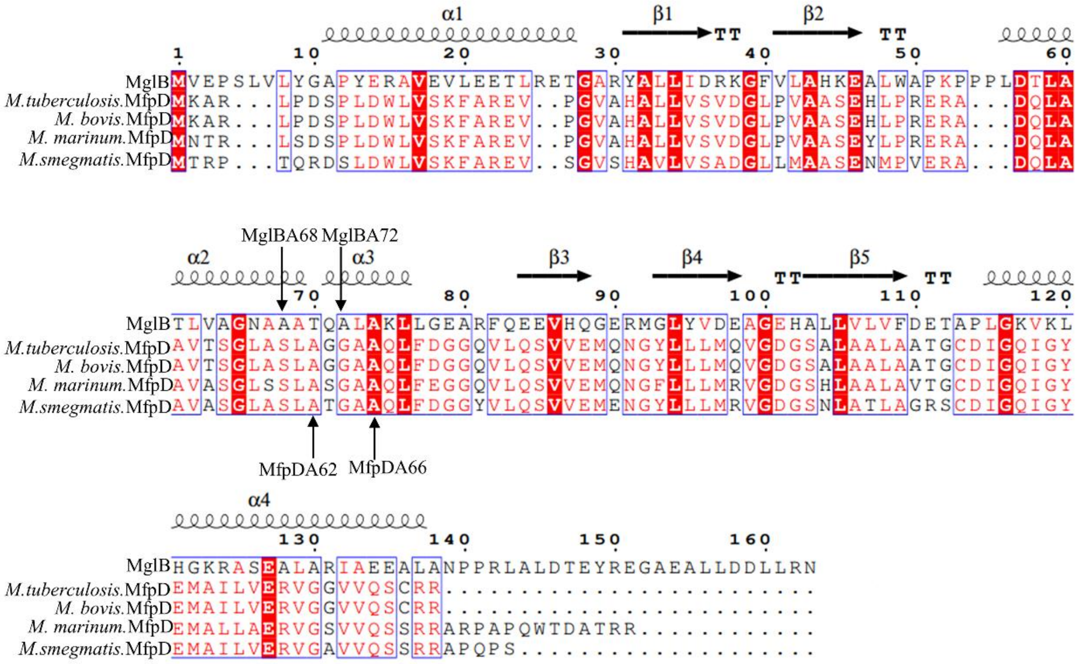

Figure S5. Multiple sequence alignment of MfpD from *Mycobacterium* and its homologs in *Myxococcus xanthus*.

Table S1

Strains, plasmids and primers used in this study

Strains	Description
M.smegmatis	Wild type *M.smegmatis* mc^{2}155
Ms_Vec	*M.smegmatis* with transformed with vector pMV-261
Ms_*mfpD*	*M.smegmatis* with transformed with vector pMV-261-*mfpD*
Δ*mfpD*	The knockout of *mfpD* in *M.smegmatis*
Δ*mfpD*_Vec	Δ*mfpD* with transformed with vector pMV-261
Δ*mfpD*::*mfpD*	Δ*mfpD* with transformed with vector pMV-261-*mfpD*
Δ*mfpB*	The knockout of *mfpB* in *M.smegmatis*
Δ*mfpB*Δ*mfpD*	The knockout of *mfpB* and *mfpD* in *M.smegmatis*
Δ*mfpB*::*mfpD*	Δ*mfpB* with transformed with vector pMV-261-*mfpD*
Δ*mfpC*	The knockout of *mfpC* in *M.smegmatis*
Δ*mfpC*Δ*mfpD*	The knockout of *mfpC* and *mfpD* in *M.smegmatis*
Δ*mfpA*	The knockout of *mfpA* in *M.smegmatis*
Δ*mfpA*Δ*mfpB*	The knockout of *mfpA* and *mfpB* in *M.smegmatis*
E.coli DH5α	Strain used in vector proliferation
E.coli BL21	Strain used in vector expression and purification
**Plasmids**	**Description**
pMV-261	A replicative plasmid used for gene expression in *M.smegmatis* and conferring kanamycin(kan) resistance

pUAB100	A plasmid used for M-PFC
pUAB200	A plasmid used for M-PFC
pUAB300	A plasmid used for M-PFC
pUAB400	A plasmid used for M-PFC
pJV53-Cpf1	A plasmid used for knockout in *M.smegmatis*
pCR-Hyg	A plasmid used for knockout in *M.smegmatis*
pET28	A replicative plasmid with His-tag and sumo-tag used for gene expression and purification in *E. coli* and conferring kanamycin(kan) resistance
**Primer**	**Sequence(5'-3')**
pMV-261-mfpD-F	AGAATTCATGACCCGTCCGACGCAG
pMV-261-mfpD-R	TAAGCTTTCAAGACGGTTGGGGCGC
crmfpDup	ATCCTGGACTGGCTGGTGTCCAAGTTCA
crmfpDdw	AGCTTGAACTTGGACACCAGCCAGTCCAGGATCT
MfpDP1	CGAACCCGCTGCCGAGGTGTCC
MfpDP2	CGCGCGGCGCGACGACTGGTCCAGGGAATCGCGCTG
MfpDP3	CAGCGCGATTCCCTGGACCAGTCGTCGCGCCGCGCG
MfpDP4	GCCACGCAGGGTCCTTCCTATCAGTT
pMV-261-F	GGCATAGGCGAGTGCTAAGAATAACG
pMV-261-R	CCCAGTCTTTCGACTGAGCCTTT
37-38(up)	AACGGCGAAATTCACGGAAACG
37-38(down)	GCAGGTAGCCGTTCTCCATCTCG
38-39(up)	GGCTACGTGCTGCAATCGGTGGT
38-39(down)	GATAACCCTGCTTCACCAAGTCCC
39-40(up)	TCGCAGCACATTTGTCCCTACC
39-40(down)	GACCGTGGTGGTGCGTTTGT
40-41(up)	AGAAGGAACACCCGACAAACGC
40-41(down)	GAACGCCGATCCATGATGCTC
RTmfpAup	AAACGGCGACGAAACGGTATG
RTmfpAdw	CGCACTCGACGAATGTGACAGG
RTmfpBup	CCCGCTAGAAGGAACACCCGACAA
RTmfpBdw	ACGGCGTGCGTGGGATGCTTTG
crmfpBup	ATGGAGATCATGCCGCTGCGGACAGAGA
crmfpBdw	AGCTTCTCTGTCCGCAGCGGCATGATCTCCATCT
MfpBP1	TGGAGAACGGCTACCTGCTGCTGA
MfpBP2	CGGGGTCAGGGTGCTCAGCACGATCTTCGTCGAAGC
MfpBP3	GCTTCGACGAAGATCGTGCTGAGCACCCTGACCCCG
MfpBP4	CGTGGTCCAGAACGTGGGGTCG
crmfpCup	ATGTCCCTACCTCTCGGCGTGGCGAGAA
crmfpDdw	AGCTTTCTCGCCACGCCGAGAGGTAGGGACATCT
MfpCP1	AAGCTTCGGTAGCATCTGCCAACGA
MfpCP2	CCTATCAGTTCGCGGCGCCGGCTCGTCGGTGCGCTC
MfpCP3	GAGCGCACCGACGAGCCGGCGCCGCGAACTGATAGG
MfpCP4	CATATGCCGCCCATACCGTTTCGTC
crmfpAup	ATACCAACTGCAGCCTGCTCGGGTCGGA

crmfpAdw	AGCTTCCGACCCGAGCAGGCTGCAGTTGGTATCT
MfpAP2	CTGCTCGCGGTGAGAAACCGTTTCGTCGCCGTTTGC
MfpAP3	GCAAACGGCGACGAAACGGTTTCTCACCGCGAGCAG
MfpAP4	CGCTGACCATCCGCGATCTCAT
1641(+132)	CGCTGGAAGATCCGTCTCAAAGC
pUAB400mfpD-F	CGGAATTCGATGACCCGTCCGACGC
pUAB400mfpD-R	Same as the pMV-261-mfpD-R
pUAB300mfpB-F	GAGGATTCGTGGCCTACGAGCACT
pUAB300mfpB-R	AAAGCTTTCAGCCGGGCAGCGGGGT
pUAB400mfpA-F	TGGAATTCGGTGCGTATAGGGGCAAA
pUAB400mfpA-R	TGAAGCTTCTAGCCGCCGTGGACGG
pET28a-mfpD-F	Same as the pMV-261-mfpD-F
pET28a-mfpD-R	Same as the pMV-261-mfpD-R
pET28a-mfpB-F	AGAATTCGTGGCCTACGAGCACTCT
pET28a-mfpB-R	Same as the puAB400mfpB-R
MfpDR19GP2	TCCAAGTTCGCCGGCGAGGTTAGCGGGGTGTCCCATGCGGTGCTGGTG
MfpDR19GP3	CACCAGCACCGCATGGGACACCCCGCTAACCTCGCCGGCGAACTTGGA
MfpDR47GP2	AGTGAGAACATGCCGGTCGAAGGCGCCGACCAGCTCGCCGCGGTG
MfpDR47GP3	CACCGCGGCGAGCTGGTCGGCGCCTTCGACCGGCATGTTCTCACT
MfpDR120P2	ATGGCGATCCTCGTCGAAGGCGTCGGCGCCGTCGTGCAGTCG
MfpDR120GP3	CGACTGCACGACGGCGCCGACGCCTTCGACGAGGATCGCCAT
MfpBG23VP2	TGTCGTCTTGCCGGCGACGAATCCCCCGCTTATCACGATCTTCGTCGA
MfpBG23VP3	TCGACGAAGATCGTGATAAGCGGGGGATTCGTCGCCGGCAAGACGACA
MfpBQ89AP2	CATGAACCAGAACCTGCGGCCACCGGGAGTGCCGAACAG
MfpBQ89AP3	CTGTTCGGCACTCCCGGTGGCCGCAGGTTCTGGTTCATG
RTtopoNup	TGTGGAGCAAGCTGGCACTGGA
RTtopoNdw	TGTTCAACGGGAAACCGTAGACGT
RTtopoMup	CATCGTCGTCACCGCCAAGC
RTtopoMdw	AAGTTGCAGCGACAGAACGTAATCC
pUAB300TopoN-F	CGGGATCCGTGAGTTACACCGCCGC
pUAB300TopoN-R	CCAAGCTTCTACTTGTTCAACGGGAA
